# Community Knowledge About the Use, Reuse, Disinfection and Disposal of Masks and Filtering Facepiece Respirators: Results of a Study Conducted in a Dermatology Clinic at the University of Naples in Italy

**DOI:** 10.1007/s10900-020-00952-3

**Published:** 2020-11-30

**Authors:** Massimiliano Scalvenzi, Alessia Villani, Angelo Ruggiero

**Affiliations:** Section of Dermatology—Department of Clinical Medicine and Surgery, University of Naples Federico II, Via Pansini 5, 80131 Napoli, Italy

**Keywords:** Facial mask, Surgical mask, FFP, N95, COVID-19

## Abstract

In Italy, as well as in almost all countries, the use of masks in public with several other measures has been an important health measure during the ongoing COVID-19 pandemic. The correct use of masks is essential, as a wrong use and disposal may increase the rate of contagious. Herein, we report a descriptive study evaluating the knowledge and use, reuse and disposal of masks in community settings. An anonymous questionnaire called MaSK (Mask uSe and Knowledge) questionnaire was developed and offered to patients referring at our dermatologic outpatient clinic. A total of 2562 full complete patients’ questionnaires were considered for the study. Our results showed that awareness and information campaigns aimed at the general population are urgently needed in order to implement a correct use of masks and limit as much as possible the infection rate.

## Introduction

In the context of the COVID-19 pandemic period, different measures have been applied in order to reduce as much as possible the spread of infection, with the use of masks as one of the most important indication given to the population. However, the supply of personal protective equipment (PPE) represented an important problem for both availability and affordability [1]. In Italy, as well as in almost all countries, the use of masks in public with several other measures (i.e. social distance) have been an important health measure during the ongoing COVID-19 pandemic [2]. Indeed, the use of face masks and filtering facepiece respirators (FFP) (i.e. N95 or FFP2 or FFP3 or equivalent with a minimum filtration efficiency of 95%) has been recommended to reduce the risk of infection [3, 4]. Furthermore, it has been showed that surgical masks reduce virus detection in large respiratory droplets and in aerosols, suggesting that surgical face masks could be used by infected patients to reduce virus transmission [4]. The correct use of masks is essential, as a wrong use and disposal may increase the rate of contagious [5]. Moreover, due to availability and affordability concerns, re-use of surgical masks and FFP has been suggested, however, a decontamination is mandatory to their re-use [1]. We developed a questionnaire called MaSK (Mask uSe and Knowledge) questionnaire to investigate the knowledge of patients about the correct use, re-use and disposal of masks and FFP used to prevent COVID-19 spread.

## Materials and Methods

This descriptive study was conducted between 1 April and 1 September, 2020. Patients referring to our outpatient dermatologic clinic and at our tele-dermatology services of the University of Naples Federico II in Italy have been consecutively enrolled in the study. A total of 2655 patients anonymously completed the questionnaires. Patients enrolled in the study were systematically offered to complete an anonymous questionnaire, after obtaining their informed consent for data collection and for the study, as well as privacy statements. The questionnaire called MaSK (Mask uSe and Knowledge) questionnaire, is a 14-items questionnaire developed at the Dermatologic Clinic of the University of Naples Federico II, with the main purpose of collecting patients’ knowledge about the use, re-use and disposal of masks and FFP used to prevent COVID-19 spread. The questionnaire was proposed to the patients during or at the end of the visit (both in person or via telemedicine service). It is composed of two sections. Section A gathered the patient’s demographic characteristics (sex, age), section B is focused on the Mask uSe and Knowledge (the type of masks and FFP respirators known, used, duration time of each type, disposal) and a direct question investigating on patients’ opinion about the use of masks. (Table 1). The study was approved by the university’s Ethics Committee.

**Table 1:** MaSK (Mask uSe and Knowledge) questionnaire

Section A
1.Sex M/F
2.Age range:
(a) under 18 years old
(b) 18–30 years old
(c) 30–50 years old
(d) 50–70 years old
(e) over 70 years old
Section B
1. Do you regularly use face masks and / or filtering facepiece respirators?
(a) Yes
(b) No
2. How many hours per day do you wear a face mask or filtering facepiece respirators?
(a) less than 1 h
(b) 1–3 h
(c) 3–5 h
(d) more than 5 h
3. Which type of mask(s) do you know? (multiple answers allowed)
(a) surgical mask
(b) FFP1
(c) FFP2
(d) FFP3
(e) N95
(f) Cloth mask
4. Which type(s) do you use most frequently? (multiple answers allowed)
(a) surgical mask
(b) FFP1
(c) FFP2
(d) FFP3
(e) N95
(f) Cloth mask
5. How long do you use a surgical mask before changing it? (if you do not use surgical masks answer on the basis of your opinion / knowledge):
(a) 4–8 h
(b) 1 day
(c) 2–5 days
(d) 1 week
(e) More than 1 week
6. How long do you use a FFP1 before changing it? (if you do not use FFP1 answer on the basis of your opinion / knowledge):
(a)10–12 h
(b) 1 day
(c) 2–5 days
(d) 1 week
(e) More than 1 week
7. How long do you use a FFP2 before changing it? (if you do not use FFP2 answer on the basis of your opinion / knowledge):
(a)10–12 h
(b) 1 day
(c) 2–5 days
(d) 1 week
(e) More than 1 week
8. How long do you use a FFP3 before changing it? (if you do not use FFP3 answer on the basis of your opinion / knowledge):
(a)10–12 h
(b) 1 day
(c) 2–5 days
(d) 1 week
(e) More than 1 week
9. When you are not wearing your mask or FFP, where do you usually put it?
(a) wrist / arm
(b) In a specific plastic bag
(c) I fold it and put in trousers pocket
(d) I always throw it away
(e) Other ( please specify)
10. After you use a mask or FFP, how do you wash or disinfect it?
(a) Only with water
(b) With specific cleansers
(c) With alcohol
(d) I never clean or disinfect masks
(e) I always throw it away
(f) Other ( please specify)
11. Where do you usually throw away your mask or FFP?
(a) In a whatever waste basket
(b) In a specific waste basket
(c) In general waste
(d) In organic waste
(e) In the paper waste
(f) Other ( please specify)
12. In your opinion, do you believe that the use of masks or FFP represents a useful tool to prevent the spread of COVID-19?
(a) Yes
(b) No

The MaSK questionnaire is composed by two section: i) Section A investigating about sex and age range; ii) Section B investigating about the use of masks (questions 1–2, 4–8), knowledge of mask types (question 3), the reuse of masks (question 9), the disinfection of masks (question 10), the disposal of masks (question 11) and patients’ opinion on the utility of masks use in prevent COVID-19 infection (question 12)

*FFP* filtering facepiece respirators

### Statistical Analysis

Data were presented as number and proportion of patients (categorical variables). The significance of the difference in patients’ answers (based on gender and age groups), was assessed by Chi-square and Fisher’s tests (alpha < 0.05), where p-values < 0.05 were considered to be statistically significant. All statistical analyses were performed using GraphPad Prism 4.0 (GraphPad Software Inc., La Jolla, CA, USA).

## Results

**Fig. 1:**
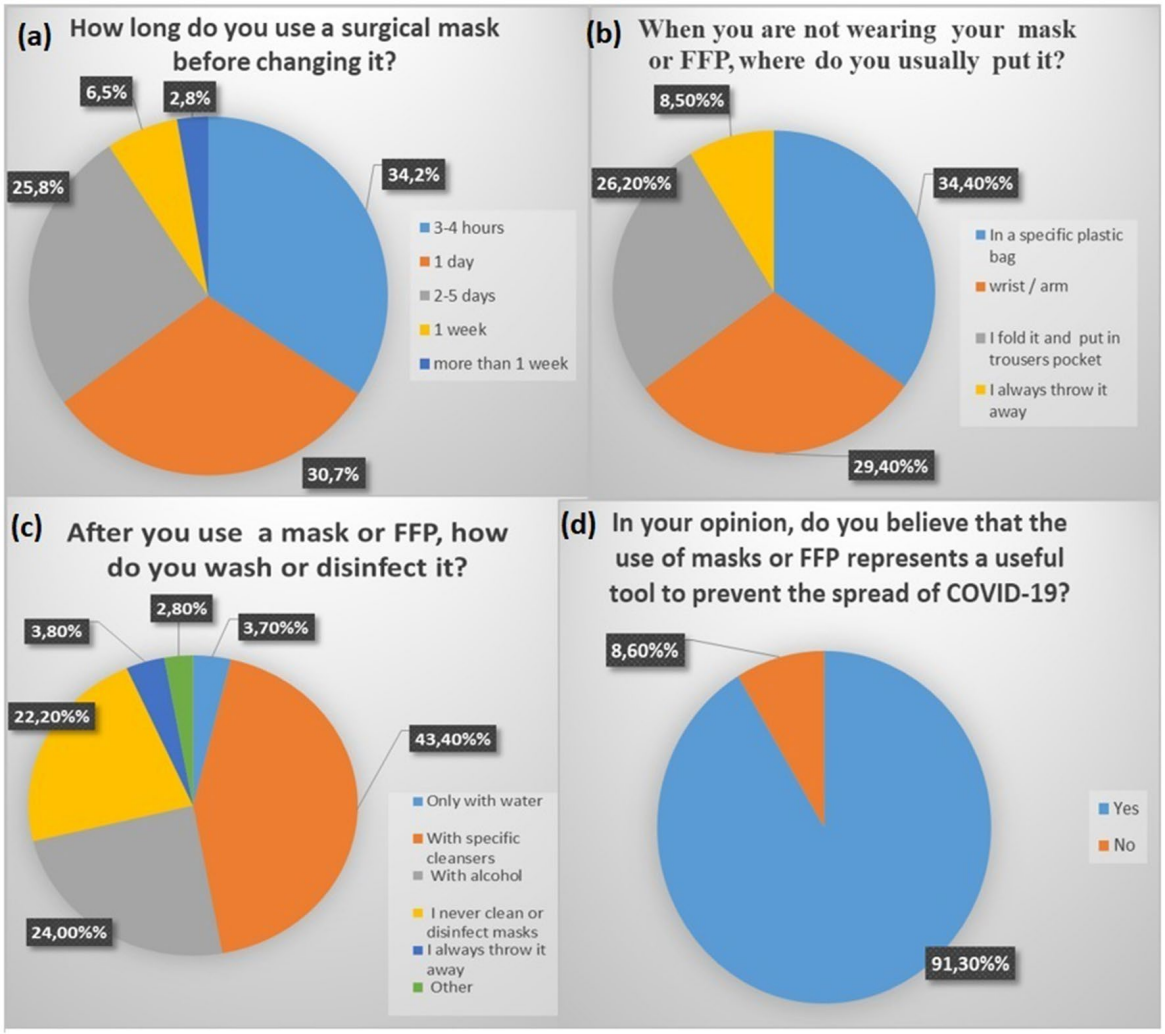
**a** Answers rate at the question “How long do you use a surgical mask before changing it?”; **b** Answers rate at the question “When you are not wearing your mask or FFP, where do you usually put it?”; **c** Answers rate at the question “After you use a mask or FFP, how do you wash or disinfect it?”; **d** Answers rate at the question “In your opinion, do you believe that the use of masks or FFP represents a useful tool to prevent the spread of COVID-19?”

A total of 2655 patients anonymously completed the questionnaires and were consecutively enrolled in the study. However, 93 questionnaires were not considered due to the lack of answers. Hence, a total of 2562 full complete patients’ questionnaires were considered for the study. The study population included 1381 (52.1%) female and 1271 (47.9%) male. The most frequent age range was 30–50 year-old (35.4%, n = 907), followed by 50–70 year-old (31.4%, n = 804), 18–30 year-old (22.6%, n = 579), over 70 year-old (7.7%, n = 197) and under 18 year-old (n = 75, 2.9%). Almost all patients (98.2%, n = 2516) declared to use regularly face masks and/or FFP, while only 1.8% (n = 46) declared to not use any masks or FFP. 42.3% (n = 1083) reported to use mask or FFP at least for 1–3 h per day, 23.8% (n = 610) reported to use PPE for 3–5 h per day, 18.3% (n = 469) reported to use PPE less than 1 h per day and 15.6% (n = 400). As regards types of masks or FFP known, the most known type was surgical masks (95.7% n = 2452), followed by cloth masks (75.8%, n = 1941), N95 (53.5%, n = 1370), FFP2 (50.4%, n = 1291), FFP1 (44.1%, n = 1130) and FFP3 (33.6%, n = 860). Surgical mask resulted the most used type of mask (80.6% n = 2065) followed by cloth masks (40.1%, n = 1027), N95 (17.5%, n = 448), FFP2 (7.8%, n = 200), FFP1 (4.1%, n = 105) and FFP3 (1.7%, n = 43). Patients reported the use of surgical mask before changing it for 3–4 h in 34.2% (n = 876) of cases, 1 day in the 30.7% (n = 786), 2–5 days in the 25.8% (n = 661), 1 week in the 6.5% (n = 166) and more than 1 week in 2.8% (n = 72). As regards the use of FFP2, patients reported the use of FFP2 before changing it for about 12 h in 21.6% (n = 533), 1 day in 20.8% (n = 533), 2–5 days in 28% (n = 717), 1 week in 21.4% (n = 548) and more than 1 week in 8.3% (n = 213). As regards the use of FFP3, patients reported the use of FFP3 before changing it for about 12 h in 18.3% (n = 469) of cases, 1 day in 18.7% (n = 479), 2–5 days in 26.5% (n = 679), 1 week in 23% (n = 589) and more than 1 week in 13.5% (n = 346) of patients. 34.4% (n = 881) declared to keep their masks in a specific plastic bag when they are not using it, 29.4% (n = 753) declared to put their masks at wrist or arm, 26.2% (n = 671) declared to fold and put masks in trousers pocket, while only 8.5% (n = 218) declared that they always throw masks away. About disinfection, 77.8% (n = 1993) patients declared to disinfect their masks or FFP. Particularly, the most common way used to disinfect masks was with specific cleansers (43.4%, n = 1112), followed by alcohol (24%, n = 615), only water (3.7%, n = 95) and among other reported ways there were hot water (1.6%, n = 41) and sun exposure (1.2%, n = 31). 22.2% (n = 569) declared that they never clean or disinfect masks, while the rest (3.8%, n = 99) declared that they always throw masks away. The majority of patients declared to throw mask and FFP away in general waste (70.5%, n = 1806), while 13.4% and 11% reported to throw masks away in a specific waste basket (n = 343) and in a whatever waste basket (n = 282). Only few patients declared to use organic (2.2%, n = 56) and paper (2%, n = 51) wastes. The last questionnaire point investigated on patients’ opinion about the use of masks or FFP, asking if they believe that these PPE may be a useful tool to prevent COVID-19 spread. Most patients (91.4%, n = 2340) recognise the utility of masks. However, a non-treasurable part of them (8.6%, n = 222) reported to believe that masks or FFP are not useful to prevent the spread of infection. As regards gender differences, male patients declared to use masks and FFP for more time if compared to female patients. Particularly, among patients re-using a mask for more than 1 day [2–5 days, 1 week and more than 1 week (a total of 899 patients)], the majority was represented by male patients [72.2% (n = 649) male patients, 27.8% (n = 250)]. Hence, in the group of patients reporting the use of masks for one day or less, 67.7% (n = 1131) were females and 32.3% (n = 539) were males. Moreover, among patients that declared to not clean their masks (22.2%, n = 569), 86.6% (n = 493) of them were male patients (13.4%, n = 76 females). Regarding the last question’s answer, among patients declaring that masks and FFP were not useful to prevent the spread of infection, 125 (56.3%) out of 222 patients were male. These differences were statistically significant (p < 0.0001). No significant differences were found about the type of masks known and used, as well as about how to throw away masks. Regarding differences in age groups, among patients re-using a mask for more than 1 day [2–5 days, 1 week and more than 1 week (a total of 899 patients)], half of them were 50–70 year-old (50.8%, n = 457), 24.2% (n = 217) were 18–30 year-old, 14% (n = 126) were 30–50 year-old, 6.7% (n = 60) were over 70 year-old and 4.3% (n = 39) were under 18 year-old. Cloth masks resulted to be more used in younger patients (under 18 and 18–30 groups) than in older groups. 604 out of 654 (under 18 and 18–30 groups) patients reported to prefer a cloth mask, representing the 58.8% of patients using cloth masks. Most of 30–50 and 50–70 year-old groups declared to not clean their masks, representing 85.5% of them (487 out of 569 patients were in these 2 groups) while the rest (14.5%) were equally divided in other groups. Regarding the last question’s answer, among patients declaring that masks and FFP were not useful to prevent the spread of infection (a total of 222 patients), 40.7% (n = 90) were 18–30, 35.7% (n = 79) were 50–70, 20.6% (n = 46) were 30–50, 2.2% (n = 5) were under 18 and 0.8% (n = 2) were over 70. These differences were statistically significant (p < 0.0001). No significant differences were found about the type of masks known, as well as about masks disposal among different groups (Fig. 1).

## Discussion

Since the COVID-19 outbreak, different measures have been applied in order to reduce the spread of infection [6]. However, to prevent a new lockdown, interventions to limit transmission are urgently needed [7]. The first and most important recommendation given to the population, has been the implementation of PPE use, associated with social distancing. Among PPE, surgical masks and FFP are the most common PPE used. Indeed, the use of masks, has been showed to reduce infections and deaths [7, 8]. Recent studies investigating the use of masks as a strategy to control the spread of infection, suggested masks as important tools during the COVID-19 pandemic in both community and health care settings [9]. Surgical face masks were introduced to protect patients from wound infection during surgical procedures. Later, these have been adopted to protect healthcare workers against infections [4]. During the COVID-19 pandemic these became the most frequent PPE adopted among community. In a recent study investigating the efficacy surgical masks in preventing the aerosols influenza transmission, surgical masks showed to be able to limit the release of the droplets and the diffusion of infection [10]. Moreover, other studies comparing the effectiveness of surgical masks and N95 masks revealed no significant difference between these PPEs [11]. FFP are personal protectors respirators covering nose and mouth against the airborne particles such as dust or infectious agents [11]. FFPs contribute in air-purifying, reducing the risk of infection diffusion [12, 13]. On average, the protection factors of FFPs have been reported to be from 11.5–15.9 times greater than those of surgical masks [14]. The filtration efficiency of FFPs may vary between 82 and 99%, depending on the different filtration grade [15]. There are different types of FFP respirators: N95, FFP2, FFP3. N95, FFP2, FFP3 apply to different standards (FFP2 and FFP3: European Committee for Standardization: EN149:2001‐A1:2009; N95:NIOSH:42 CFR Part 84). N95 masks are equivalent to FFP in European countries with an efficacy grade of 95%. The use of N95 and FFP2 has been constantly growing during the pandemic period particularly among healthcare workers. Even if most laboratory data report that N95 have a protective advantage over surgical masks, a recent meta-analysis showed that available data are not sufficient to determine definitively whether N95 are superior to surgical masks in terms of protection against respiratory infections in clinical settings [16]. FFP3 masks, though not as widely used, showed 99% efficacy of filtration grade. This type of mask is less used in community settings, while it results most commonly used among healthcare workers. Indeed, FFP3, instead of N95/FFP2, has been recommended when exposed to aerosol‐generating procedures in COVID‐19 patients [17]. Furthermore, FFP3 can be an important and essential part of PPE during dental procedures [18]. As regards community use of FFP3, this class represents the least mask used among population, for both the higher cost and difficulty in finding it. During the lockdown period, the worldwide use of masks resulted in severe shortages of PPE, including surgical and FFP masks, leading some governments to suggest the use of cloth face masks as a last resort when standard PPEs are unavailable [19]. Data about the filtration efficiency of cloth masks are still poor. Recent studies suggested filtration effectiveness between 3 and 95% [20]. However, in order to prevent PPE shortage crisis for healthcare workers, health authorities recommended the use cloth masks [21]. On the other hand, cloth masks may provide a well grade of protection if correctly designed and used. Indeed, multilayer cloth masks and made of water-resistant fabric may represent an effective PPE in community settings [22]. Cloth mask should not be suggested for healthcare workers, who should preferentially use surgical or FFP masks. Hence, cloth mask use may be proposed in community settings in case of unavailability of other facial masks [22]. In the pandemic period, one of the last resort strategies during mask shortages has been represented by masks decontamination and reuse [23]. Indeed, many strategies to disinfect and to sterilize masks have been suggested to the population in order to be able to reuse masks. Different ways with different results have been proposed to re-use masks (both surgical and FFP) in healthcare settings, such as via autoclaving (FFP) [24], germicidal ultraviolet light, vaporised hydrogen peroxide or dry heat [25]. Among community, the most frequent methods used have been the decontamination with hot water and the chemical decontamination (with ethanol or specific cleanser). Indeed, it has showed that a constant temperature over 56 °C for 30 min may kill COVID-19 virus can [26]. Hot water approach compared with other proposed decontamination methods for masks is more suitable for people to perform at home without the use of additional solvents or high-tech equipment, showing to be a safe and effective method to reuse masks [27]. As regards ethanol decontamination method, it has been showed that ethanol treatment may damage masks, resulting in the filter efficiency decrease occurred in surgical mask and the N95 respirator. [28]. However, ethanol decontamination is still a common method used among population in community settings. The massive generations of contaminated face masks may cause environmental concerns. After being used, face masks should be disposed as hazardous and infectious medical waste [29]. In daily practice, many procedures of waste disposal may be used, such as waste disposal into block-waste containers or specific street waste containers [29]. Furthermore, users should be informed and trained on how to correctly pack, collect and dispose masks, considering masks as contaminated waste. In community settings, on the basis of the criteria indicated by the legislation (Report ISS COVID-19 n. 26/2020), the masks and gloves produced by domestic activities, listed in Chapter 20 of the EER, can be classified as “urban waste” (identifiable by the EER 200301 code). Our study investigated on community knowledge, use, reuse and disposal of masks and FFP respirators using MaSK (Mask uSe and Knowledge) questionnaire, an anonymous questionnaire offered to patients referring to our outpatient clinic. Almost all of the patients (98.2%, n = 2516) declared to use regularly face masks and/or FFP, while only 1.8% (n = 46) declared to not use any masks or FFP. Our results showed that surgical and cloth masks are the most common masks used in community settings, showing a large knowledge also of other types of masks which resulted to be less used in community settings. Recent study on surgical masks duration effectiveness reported a decrease in filtration effectiveness after 4-h of wearing time [30]. However, both because of the cost and the difficulty in finding masks during the pandemic, masks are often reused. Indeed, we found that only 34.2% (n = 876) and 30.7% (n = 786) of patients reported the use of masks for 4 h and for 1 day respectively, while 35.1% reported to use of masks for more than 1 day [25.8% (n = 661) 2–5 days; 6.5% (n = 166) 1 week; 2.8% (n = 72) more than 1 week]. Moreover, 22.2% (n = 569) declared that they never clean or disinfect masks, most of them (85.5%) were in the 30–50 year-old and 50–70 year-old groups. Only 34.4% (n = 881) declared to keep their mask in a specific plastic bag when they are not using it, while 29.4% (n = 753) declared to put their mask at wrist or arm, 26.2% (n = 671) declared to fold and put mask in trousers pocket. These common and non-sterile ways to keep masks may obviously lead to an increased risk of masks contamination. As regards where to throw used masks, 70.5%, (n = 1806) and 13.4% (n = 343) declared to throw masks away in general waste and in a specific waste basket respectively, while the rest reported to use other type of wastes [11% (n = 282) a whatever waste, 2.2% (n = 56) organic waste and 2% paper (n = 51)]. To the best of our knowledge this is the first study evaluating masks use in community setting, non-specific for healthcare settings. Hence, our data suggest that there are still too many people which does not know how to correctly use, reuse and disposal masks. Particularly we found that most of our study population reuse masks for a long time without using any disinfection or using methods which may decrease the filtration efficacy of masks if repeated too many times. Furthermore, only 34.4% (n = 881) declared to keep their masks in a specific plastic bag when they are not using it, while the rest declared to put their mask at wrist or arm (29.4%), to fold and put mask in trousers pocket (26.2%), with the high related risk of mask contamination. Moreover, a harmful reported result was the rate of people (8.6%) believing that masks are not useful to prevent the spread of infection. Disinformation, wrong convictions and knowledge related to masks use and reuse, may lead to an increase risk of infection among community in this post lockdown period. Hence, awareness and information campaigns aimed at general population are urgently needed in order to implement a correct use of masks and limit as much as possible the infection rate.

## Limitations

Our study population is composed by patients referring to our outpatient clinic in a southern region of Italy, hence, our data may not reflect the general Italian population.

## References

[1] Ludwig-Begall LF, Wielick C, Dams L (2020). The use of germicidal ultraviolet light, vaporised hydrogen peroxide and dry heat to decontaminate face masks and filtering respirators contaminated with a SARS-CoV-2 surrogate virus. Journal of Hospital Infection.

[2] Rab S, Javaid M, Haleem A, Vaishya R (2020). Face masks are new normal after COVID-19 pandemic. Diabetology & Metabolic Syndrome.

[3] World Health Organization (WHO) (2020). Rational use of personal protective equipment for coronavirus disease 2019 (COVID-19).

[4] Leung NHL, Chu DKW, Shiu EYC (2020). Author Correction: Respiratory virus shedding in exhaled breath and efficacy of face masks. Nature Medicine.

[5] Kumar J, Katto MS, Siddiqui AA (2020). Knowledge, attitude, and practices of healthcare workers regarding the use of face mask to limit the spread of the new coronavirus disease (COVID-19). Cureus.

[6] Marasca C, Ruggiero A, Annunziata MC, Fabbrocini G, Megna M (2020). Face the COVID-19 emergency: Measures applied in an Italian Dermatologic Clinic. Journal of the European Academy of Dermatology and Venereology.

[7] Worby CJ, Chang HH (2020). Face mask use in the general population and optimal resource allocation during the COVID-19 pandemic. Nature Communications.

[8] Tirupathi R, Bharathidasan K, Palabindala V, Salim SA, Al-Tawfiq JA (2020). Comprehensive review of mask utility and challenges during the COVID-19 pandemic. Le Infezioni in Medicina.

[9] MacIntyre CR, Chughtai AA (2020). A rapid systematic review of the efficacy of face masks and respirators against coronaviruses and other respiratory transmissible viruses for the community, healthcare workers and sick patients. International Journal of Nursing Studies.

[10] Milton DK, Fabian MP, Cowling BJ, Grantham ML, McDevitt JJ (2013). Influenza virus aerosols in human exhaled breath: particle size, culturability, and effect of surgical masks. PLoS Pathogens.

[11] Tcharkhtchi A, Abbasnezhad N, Zarbini Seydani M, Zirak N, Farzaneh S, Shirinbayan M (2020). An overview of filtration efficiency through the masks: Mechanisms of the aerosols penetration. Bioactive Materials.

[12] Leung NH (2020). Respiratory virus shedding in exhaled breath and efficacy of face masks. Nature Medicine.

[13] Konda A, Prakash A, Moss GA, Schmoldt M, Grant GD, Guha S (2020). Aerosol filtration efficiency of common fabrics used in respiratory cloth masks. ACS Nano.

[14] Lee SA, Hwang DC, Li HY, Tsai CF, Chen CW, Chen JK (2016). Particle size-selective assessment of protection of European Standard FFP respirators and surgical masks against particles-tested with human subjects. Journal of Healthcare Engineering.

[15] Jeong SB, Ko HS, Seo SC, Jung JH (2019). Evaluation of filtration characteristics and microbial recovery rates of commercial filtering facepiece respirators against airborne bacterial particles. Science of the Total Environment.

[16] Smith JD, MacDougall CC, Johnstone J, Copes RA, Schwartz B, Garber GE (2016). Effectiveness of N95 respirators versus surgical masks in protecting health care workers from acute respiratory infection: A systematic review and meta-analysis. CMAJ.

[17] Regli A, Sommerfield A, von Ungern-Sternberg BS (2020). The role of fit testing N95/FFP2/FFP3 masks: a narrative review. Anaesthesia.

[18] Rollings L (2020). FFP3 respirator face fit testing—What is it all about?. British Dental Journal.

[19] Roberge RJ, Roberge MR (2020). Cloth face coverings for use as facemasks during the coronavirus (SARS-CoV-2) pandemic: What science and experience have taught us. Disaster Medicine and Public Health Preparedness.

[20] Sunjaya AP, Morawska L (2020). Evidence review and practice recommendation on the material, design and maintenance of cloth masks. Disaster Medicine and Public Health Preparedness.

[21] Beesoon S, Behary N, Perwuelz A (2020). Universal masking during COVID-19 pandemic: Can textile engineering help public health? Narrative review of the evidence. Preventive Medicine.

[22] Chughtai AA, Seale H, Macintyre CR (2020). Effectiveness of cloth masks for protection against severe acute respiratory syndrome coronavirus 2. Emerging Infectious Diseases.

[23] Bhattacharjee S, Bahl P, Chughtai AA, MacIntyre CR (2020). Last-resort strategies during mask shortages: Optimal design features of cloth masks and decontamination of disposable masks during the COVID-19 pandemic. BMJ Open Respiratory Research.

[24] Czubryt MP, Stecy T, Popke E (2020). N95 mask reuse in a major urban hospital—COVID-19 response process and procedure. Journal of Hospital Infection.

[25] Ludwig-Begall LF, Wielick C, Dams L (2020). The use of germicidal ultraviolet light, vaporised hydrogen peroxide and dry heat to decontaminate face masks and filtering respirators contaminated with a SARS-CoV-2 surrogate virus. Journal of Hospital Infection.

[26] National Health Commission of the People’s Republic of China. [Prevention and control program of COVID-19 (4th edition)]. Beijing: General Office of National Health Commission of the People’s Republic of China; 2020 Feb 6 [cited 2020 May 1]. Retrieved from from: http://www.nhc.gov.cn/jkj/s3577/202002/573340613ab243b3a7f61df260551dd4/files/c791e5a7ea5149f680fdcb34dac0f54e.pdf. Chinese.

[27] Wang D, Sun BC, Wang JX (2020). Can masks be reused after hot water decontamination during the COVID-19 pandemic?. Engineering (Beijing)..

[28] Grinshpun SA, Yermakov M, Khodoun M (2020). Autoclave sterilization and ethanol treatment of re-used surgical masks and N95 respirators during COVID-19: Impact on their performance and integrity. Journal of Hospital Infection.

[29] Jung S, Lee S, Dou X, Kwon EE (2021). Valorization of disposable COVID-19 mask through the thermo-chemical process. Chemical Engineering Journal.

[30] Barbosa MH, Graziano KU (2006). Influence of wearing time on efficacy of disposable surgical masks as microbial barrier. Brazilian Journal of Microbiology.

